# Aberrant early visual neural activity and brain-behavior relationships in anorexia nervosa and body dysmorphic disorder

**DOI:** 10.3389/fnhum.2015.00301

**Published:** 2015-06-02

**Authors:** Wei Li, Tsz M. Lai, Sandra K. Loo, Michael Strober, Iman Mohammad-Rezazadeh, Sahib Khalsa, Jamie Feusner

**Affiliations:** ^1^Department of Neuroscience, University of California, Los AngelesLos Angeles, CA, USA; ^2^Department of Psychiatry, University of California, Los AngelesLos Angeles, CA, USA

**Keywords:** anorexia nervosa, body dysmorphic disorder, electroencephalography, visual processing, dorsal ventral streams

## Abstract

**Background:** Body dysmorphic disorder (BDD) and anorexia nervosa (AN) share the clinical symptom of disturbed body image, which may be a function of perceptual distortions. Previous studies suggest visual or visuospatial processing abnormalities may be contributory, but have been unable to discern whether these occur early or late in the visual processing stream. We used electroencephalography (EEG) and visual event related potentials (ERP) to investigate early perceptual neural activity associated with processing visual stimuli.

**Methods:** We performed EEG on 20 AN, 20 BDD, 20 healthy controls, all unmedicated. In order to probe configural/holistic and detailed processing, participants viewed photographs of faces and houses that were unaltered or filtered to low or high spatial frequencies, respectively. We calculated the early ERP components P100 and N170, and compared amplitudes and latencies among groups.

**Results:** P100 amplitudes were smaller in AN than BDD and healthy controls, regardless of spatial frequency or stimulus type (faces or houses). Similarly, N170 latencies were longer in AN than healthy controls, regardless of spatial frequency or stimulus type, with a similar pattern in BDD at trend level significance. N170 amplitudes were smaller in AN than controls for high and normal spatial frequency images, and smaller in BDD than controls for normal spatial frequency images, regardless of stimulus type. Poor insight correlated with lower N170 amplitudes for normal and low spatial frequency faces in the BDD group.

**Conclusions:** Individuals with AN exhibit abnormal early visual system activity, consistent with reduced configural processing and enhanced detailed processing. This is evident regardless of whether the stimuli are appearance–or non-appearance-related, and thus may be a reflection of general, early perceptual abnormalities. As N170 amplitude could be a marker of structural encoding of faces, lower values may be associated with perceptual distortions and could contribute to poor insight in BDD. Future studies may explore visual ERP measures as potential biomarkers of illness phenotype.

## Introduction

Individuals with body dysmorphic disorder (BDD) are preoccupied with perceived defects in their appearance, which are not noticeable or are slight to others (American Psychiatric Association, 2013). They subsequently experience significant distress, disability, and functional impairment, often accompanied by depression and suicidality (Phillips, 2005). In addition, they are often delusional in their beliefs (Eisen et al., 2006), and frequently present to plastic surgeons and dermatologists instead of mental health clinicians. BDD affects approximately 1–2% of the population (Faravelli et al., 1997; Bienvenu et al., 2000; Otto et al., 2001; Rief et al., 2006), yet is still under-studied and under-recognized.

Individuals with anorexia nervosa (AN) also have similar body image distortions, although by DSM definition this relates principally to their body weight or shape (American Psychiatric Association, 2013). Individuals with AN are often convinced that they are overweight and appear “fat,” despite significant evidence to the contrary. They then restrict their caloric intake through self-starvation, which can lead to severe malnutrition, emaciation, and in some cases death (Sullivan, 1995).

Perceptual distortions of appearance may therefore be a cardinal feature across AN and BDD. fMRI studies using own-face (Feusner et al., 2010), other-face (Feusner et al., 2007), and house stimuli (Feusner et al., 2011) all found abnormalities in primary and/or secondary visual processing systems in BDD, particularly for image types that selectively conveyed configural and holistic information. Similar experiments have not been conducted in AN, although several neuroimaging studies suggest abnormal brain activation when visually processing body images (Wagner et al., 2003; Uher et al., 2005; Sachdev et al., 2008). Multiple studies additionally suggest imbalances in local (detail) vs. global processing in AN (56-61). Moreover, a study investigating the body inversion effect found AN individuals had deficits in discrimination of upright body images, suggesting deficits in configural processing (Urgesi et al., 2013).

Naturalistic visual stimuli, the most studied of which are faces, engage visual processing on multiple levels related to the type of information extracted (Bruce and Young, 1986). Perceptual inputs are analyzed to extract simple features, which are then combined to construct a structural model that can be compared with faces in memory. Two types of visual information, configural and featural, travel through dorsal and ventral visual streams, respectively (Goodale and Milner, 1992). Configural processing can be conceptualized as sensitivity to first order relations (the relative positions of the features), holistic processing of these features into a gestalt, and sensitivity to the relations between the features (such as distance between features) (Maurer et al., 2002). With configural processing, parts are not individually represented but instead recognized as “templates” (Tanaka and Farah, 1993). Featural processing can be conceptualized as the componential analysis of features that can be measured independently from each other, are local in their spatial extent, and are marked by discontinuities (Bartlett et al., 2003). This is also known as local part-based or fragmented-based processing (Schwaninger et al., 2002).

Our working model of visual processing dysfunction in BDD and AN is a primary deficit in configural/holistic processing (Li et al., 2013). This may result in a secondary, “inappropriate,” reliance on featural/part-based processing, which is utilized in situations in which healthy controls normally deploy configural/holistic processing. This may result in a conscious perception dominated by featural/part-based information (details), as a result of a diminished configural/holistic template to aid in integration. fMRI experiments studying BDD (Feusner et al., 2010, 2011, 2007) corroborate this model of visual processing dysfunction. A limitation of these prior fMRI studies (due to limited temporal resolution) is that it remains unclear if abnormal performance/brain activation patterns are primarily the result of aberrant early visual cortex activity or are the result of modulation from prefrontal and/or limbic systems. Electroencephalography (EEG) is better suited to discern this, as it can characterize fast changing neuronal dynamics that are not possible with fMRI. To date there have been no studies that have investigated early electrophysiological components in response to face or house processing in AN or BDD.

Evidence from a neuroimaging study in BDD, using own- and other-faces (Feusner et al., 2010) suggests dysfunction in early visual systems, including early extrastriate cortex. A similar study using houses stimuli also demonstrated abnormal visual system activation, although in later regions in the visual stream (lingual and parahippocampal gyri) (Feusner et al., 2011). We used face and house stimuli to probe the early visual systems in AN and BDD. Event related potential (ERP) components in response to faces have been well-studied in healthy controls, and facial flaws are a common concern in BDD. Houses provide a figure similar in complexity to faces with neutral salience. (Bodies stimuli, although more relevant to appearance concerns for AN subjects, were not used as their P100 and N170 components are not as well-studied or characterized as for faces or houses).

## EEG: P100 and N170 event related potential components

The P100 and N170 are visual processing components evoked by presentations of faces and objects. The P100 is the first positive visual evoked potential apparent 80–120 ms post stimulus (Spehlmann, 1965; Herrmann et al., 2005). A study that measured the P100 amplitudes to images of faces and houses, filtered to include only certain spatial frequencies, found the P100 was preferentially larger for low spatial frequency faces/houses and smallest for high spatial frequency faces/houses (Nakashima et al., 2008). Thus, the P100 may index early configural processing.

The N170 is a large negative component that is robustly evoked by face stimuli (Bentin et al., 1996; Rossion et al., 2000), although it also shows varying degrees of activation by other stimuli such as houses, cars, and other objects. It is most prominent in occipito-temporal electrodes, and occurs about 150–180 ms post-stimulus. It may reflect both configural and featural processing (Bentin et al., 1996; Sagiv and Bentin, 2001). Bentin et al. (1996) found the N170 was larger in response to eyes presented in isolation compared to full faces, suggesting the N170 was responsive to featural processing. However, another study found that face representations that require only configural processing generate similar N170 amplitudes as normal photographs of faces (Sagiv and Bentin, 2001). These results can be reconciled by the observation that in most situations faces are primarily processed configurally, whereas analytic, featural processing requires a greater recruitment of neuronal populations, as reflected in a larger N170. Campanella (Campanella et al., 2006) found diminished P100 and N170 components in response to faces in schizophrenia patients; the decreased amplitudes were ascribed to deficits in configural processing, which converges with results from several other studies (Deruelle et al., 1999; Streit et al., 2001; Herrmann et al., 2004; Javitt, 2009; Urgesi et al., 2013). Our model, in which the primary abnormality is reduced configural processing in BDD and AN, predicts a similar pattern (although perhaps not the same degree) of abnormal EEG responses.

## Spatial frequencies and their relation to configural/featural processing

In our Faces and Houses Tasks, we probed individuals' configural and featural processing by using low-pass (LSF) and high-pass (HSF) spatial frequency-filtered visual images, respectively, as has been performed previously in healthy controls using EEG (Pourtois et al., 2005; Halit et al., 2006). Early vision filters images at multiple spatial scales, tuned to different bandwidths of spatial frequencies. Discrimination and detection of simple sine wave patterns are predicted by the contrast of their individual component spatial frequencies, implying the visual system decomposes the patterns with spatial frequency filters (Campbell and Robson, 1968). Marr (1982) proposed that the visual system uses a multiscale representation of the image, constructing a stable, quick, coarse gestalt that is later fleshed out with fine-scale information.

It has been postulated that different levels of spatial frequencies in images convey different types of information for visual processing. LSF images convey information about coarse holistic features such as pigmentation or shading (Morrison and Schyns, 2001) while HSF images convey information about contours and edges. Neurons in primary visual cortex dedicate their first transient responses to processing LSF sinusoidal gratings and later shift their tuning curves to finer information (HSF gratings) (Bredfeldt and Ringach, 2002). Psychophysical evidence indicates that LSF gratings are resolved faster than their HSF analogs (Gish et al., 1986; Parker and Dutch, 1987). Finally, LSF faces have larger holistic effects compared to HSF faces for the whole-part advantage and composite face paradigms (Goffaux and Rossion, 2006).

Previous functional neuroimaging studies of visual processing in BDD used images filtered to LSF and HSF to selectively activate configural and featural processing, respectively (Feusner et al., 2007, 2011). We analyzed the ERP responses to these images to draw inferences about abnormalities in configural or featural visual processing in AN and BDD. We also included the unaltered (“Normal Spatial Frequency,” or NSF) images as they should engage both configural and featural processing.

## Hypotheses

We hypothesized that AN and BDD individuals would demonstrate abnormal early configural processing deficits along with greater reliance on detailed strategies, as both experience appearance-related concerns that could be attributed to perceptual distortions. Thus, we expected lower P100 and N170 amplitudes for AN and BDD relative to controls for normal and low detail faces, which would reflect deficiencies in configural processing. Similarly, we expected delayed N170 latencies for AN and BDD relative to controls for normal and low detail faces, due to secondary, excessive reliance on detailed strategies, which are slower than configural strategies (Goodale and Milner, 1992). We predicted the same patterns for house stimuli; although the previous study in BDD found abnormalities in later visual stream regions, we predicted that similar, earlier aberrant electrophysiological components as for faces might feed forward to contribute to later diminished activation. Abnormal ERP components for house stimuli would therefore reflect general, early visual system deficiencies that are not limited to appearance-related stimuli.

We also predicted that abnormalities in early visual processing associated with these ERP components would be associated with the clinical variable of poor insight, as aberrant perception would make it difficult for one to refute what they see, even in the presences of contrary evidence. Supporting this, a previous diffusion tensor imaging (DTI) study in BDD found associations between insight and white matter tracts connecting visual systems with emotion and memory systems (Feusner et al., 2014). We hypothesized that for the LSF and NSF images there would be an association between lower insight and lower amplitudes (N170 and P100) and longer latencies (N170) in BDD and AN.

## Methods and materials

### Participants

We enrolled 20 individuals meeting DSM-IV-TR criteria for BDD, 20 with AN, and 20 age- and gender-matched healthy controls (see Table 1). All participants were between ages 18–30 and all were unmedicated. Each BDD participant received a clinical evaluation by Jamie Feusner, who has clinical expertise in BDD. Each AN participant received a clinical evaluation by Michael Strober or Sahib Khalsa, or who have clinical expertise in AN. We used the Mini International Neuropsychiatric Inventory (MINI) to determine comorbid diagnoses (Sheehan et al., 1998). Severity of other psychiatric symptoms was measured using validated clinical scales: the Hamilton Anxiety Rating Scale (HAMA) (Hamilton, 1960), the Brown Assessment of Beliefs scale (BABS, measuring insight about perceived defects and psychiatric illness) (Eisen et al., 1998), and the Montgomery-Asberg Depression Rating Scale (MADRS) (Williams and Kobak, 2008). BDD participants received the BDD version of the Yale–Brown Obsessive–Compulsive Scale (BDD-YBOCS) (Phillips et al., 1997), and AN participants received the Eating Disorder Examination V16.0D (EDE) (Fairburn et al., 2008).

**Table 1:** **Demographics and Psychometrics for AN, BDD, and healthy control (HC) participants**.

	**Anorexia Nervosa (AN)**	**Body Dysmorphic Disorder (BDD)**	**Healthy Control (HC)**	***p*-values**
N	20	20	20	
Gender (F/M)	18/2	18/2	18/2	
Age	23.40 ± 3.22	24.60 ± 5.13	22.55 ± 4.02	*F* = 1.2, *p* = 0.31
Highest Grade Completed	15.00 ± 2.11	16.05 ± 3.47	14.38 ± 2.51	*F* = 1.83, *p* = 0.171
BDD-YBOCS (BDD) or EDE (AN) Score	2.48 ± 1.25	29.05 ± 5.38	-	
HAMA	7.30 ± 6.69^a^	10.20 ± 7.21^a^	2.05 ± 1.73^b^	*F* = 10.27, *p* < 0.001
MADRS	7.75 ± 9.10^a^	14.25 ± 7.56^a^	0.95 ± 1.32^b^	*F* = 18.74, *p* < 0.001
BABS	11.53 ± 5.71^a^	14.95 ± 3.35^b^	N/A	t = 2.30, *df* = 38, *p* = 0.027

a,b *Different superscript letters indicate significant pairwise differences from post-hoc t tests at p < 0.05*.

#### BDD inclusion/exclusion criteria

The UCLA Institutional Review Board approved this study. Written informed consent was obtained from all participants. Unmedicated individuals who met criteria for BDD as determined by the BDD Diagnostic Module (32), modeled after the DSM-IV, and who scored ≥20 on the BDD-YBOCS were eligible.

#### AN inclusion/exclusion criteria

AN participants were unmedicated and were required to be weight-restored (BMI of ≥18.5); however, they must have previously met full DSM-IV criteria for AN. We chose to study weight-restored AN individuals to avoid confounds of starvation on brain activity. Eligible participants also had to meet all other current criteria for AN on the MINI, except for amenorrhea.

#### HC inclusion/exclusion criteria

HC could not meet any criteria for Axis I disorders, including substance use disorders, on the MINI.

#### Inclusion/exclusion criteria for all participants

Participants were free from psychoactive medications for at least 8 weeks prior to entering the study. All had normal or corrected visual acuity, as verified by Snellen eye chart. Exclusion criteria included other concurrent Axis I disorders aside from major depressive disorder, dysthymia, panic disorder, social phobia, or generalized anxiety disorder, as mood and anxiety disorders are frequently comorbid in this population (Hollander et al., 1993; Kennedy et al., 1994; Veale et al., 1996; Perugi et al., 1997; Zimmerman and Mattia, 1998; Gunstad and Phillips, 2003; Phillips et al., 2006a,b; Ruffolo et al., 2006; Swinbourne and Touyz, 2007).

### Face and house-matching tasks

There were four categories of face and house stimuli: HSF, non-filtered (normal spatial frequency–NSF), and LSF; and non-filtered circles/ovals (for the faces task) or squares/rectangles (for the houses task) as controls for behavioral responses. For faces, we used digitized gray-scale photographs of male and female faces. The faces, validated for neutral emotional expression, came from the Macbrain database, Facial Emotional Stimuli, the University of Pennsylvania and the Psychological Image Collection at Stirling. We then filtered these images to various spatial frequencies as previously described (Feusner et al., 2007).

**Figure 1:**
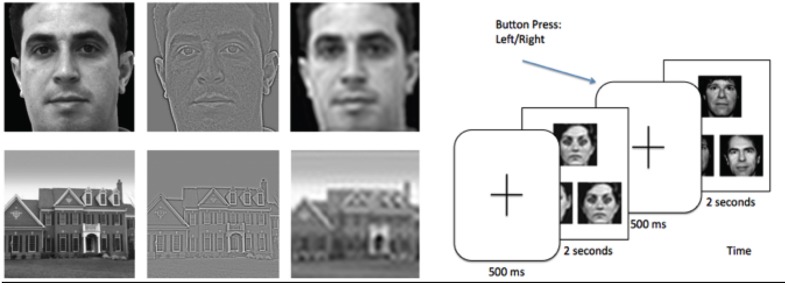
**Left: Example of stimuli used in the experiment, filtered to normal, high, and low spatial frequencies**. **Right:** Task paradigm, consisting of a face or house matching task.

After a 500 ms crosshair presentation, subjects pressed a button corresponding to which of two images match the target image in the top half of the screen, with image duration of 2 s. (see Figure 1) (Feusner et al., 2007). There were 72 trials for each category (HSF, NSF, LSF, and shapes), and spatial frequencies were not mixed within each trial. Each face or house had size 8.5 × 8.5 cm, and subtended a visual angle of 4.8°.

### EEG acquisition

All subjects were seated 1 m away from the screen. EEG data were recorded using a high density 256-channel Geodesic Hydrocel Sensor Net (Electrical Geodesics, Inc.) with a sampling rate of 250 Hz in a copper shielded room that was dimly lit. Between experiments, we checked to make sure electrode impedances were below 50 kΩ. Data preprocessing included bandpass filtering from 0.1 to 30 Hz for visual ERPs.

#### Segmentation

Data were segmented 200 ms before and 500 ms after the presentation of the stimulus for face, and house matching tasks.

#### Artifact detection

Eye blink and movement artifacts were extracted and removed using temporal Independent Component Analysis (ICA) in EEGLAB (Delorme and Makeig, 2004). Segments with > 10 bad channels were removed and interpolated from neighboring electrodes. In addition, channels and segments were visually inspected to account for artifacts missed through automatic detection. We used an interpolation algorithm to reconstruct channels marked as bad by the artifact detection algorithms. Segments were then averaged across each stimuli condition, and grand averaged over all subjects. Event related potentials (ERP) were baseline-corrected using the 200 ms baseline prior to the stimulus onset for correction. All channels were referenced to an average reference of all electrodes except electrooculography (EOG).

### Statistical analyses

#### Behavioral analyses

Accuracy (% correct) and mean correct response times were computed for each condition. We submitted data from the faces and houses task to a mixed measures ANOVA with Group (AN, BDD, or controls) as a between-groups factor and spatial frequency (high, normal, low) and stimulus type (faces, houses) as within-subjects factors. We submitted data from our shapes stimuli to a separate mixed measures ANOVA with stimulus type (circles and squares) as a within-subjects factor and Group (AN, BDD, or controls) as a between-groups factor. (There was no spatial frequency factor for the shapes, as they were not spatial frequency filtered.) We removed two outliers, defined as cases more than 1.5 times the interquartile range (above or below the 75 or 25th percentile, respectively) on stem and leaf plots in SPSS.

#### Electrophysiology

Amplitudes and latencies for P100 and N170 components were measured at a group of right hemispheric occipito-temporal electrodes (TP8, TP10, P8, PO8, P6 CP6, P10); these electrodes were chosen based on previous studies showing the N170 signal is stronger in the right hemisphere versus the left (Bentin et al., 1996; Rossion et al., 2003). Amplitudes were quantified for each condition as the peak voltage measured within 60 ms windows centered on 100 and 170 ms for the P100 and N170, respectively. Peak latency was measured as the latency at this peak voltage. These amplitudes and latencies were then submitted to a three way mixed measures ANOVA with group (AN, BDD, controls) as the between groups factor and spatial frequency (high, normal, low) and stimulus type (faces, houses) as the within group repeated measures factor. Because we were interested in group differences, we used estimated marginal means of any significant effects involving group (group, group by spatial frequency, group by stimulus type, and group by stimulus type by spatial frequency) and, following Fisher's LSD procedure, pairwise *t*-tests (uncorrected) to explore for differences between groups.

We performed Pearson's correlations between BABS scores and LSF and NSF component measures (N170 latency/amplitude, P100 amplitude) for faces and houses, with a significance level set at *p* < 0.05, one-tailed, Bonferroni-corrected.

## Behavioral results

### Reaction time

On the faces/houses tasks, there was a significant spatial frequency effect [F_(2, 53)_ = 257.61, *p* < 0.001], but no significant group [*F*_(2, 53)_ = 1.77, *p* = 0.18), stimulus type [*F*_(1, 53)_ = 0.004, *p* = 0.95], spatial frequency by group [*F*_(4, 108)_ = 0.58, *p* = 0.68], stimulus type by group [*F*_(2, 53)_ = 0.64, *p* = 0.53], spatial frequency by stimulus type (*F*_(2, 53)_ = 2.22, *p* = 0.12], or stimulus type by spatial frequency by group effects [*F*_(4, 108)_ = 0.87, *p* = 0.49]. To follow up on the significant spatial frequency effect, we performed pairwise comparisons among spatial frequencies. There were significantly shorter reaction times for the LSF than the NSF (mean difference = 33 ms, *p* < 0.001) or HSF (mean difference = 152 ms, *p* < 0.001) images, while there were significantly shorter reaction times for NSF than HSF (mean difference = 119 ms, *p* < 0.001) images.

On the shapes stimuli, there were no significant stimulus type [*F*_(1,54)_ < 0.001, *p* = 0.99], group [*F*_(2, 54)_ = 1.27, *p* = 0.29], or stimulus type by group [*F*_(2, 54)_ = 1.36, *p* = 0.27] effects for reaction time.

### Accuracy

All groups performed accurately on both face and house tasks as well as the shapes control stimuli (>95% on all tasks). On the faces/houses tasks, there was a significant spatial frequency effect [*F*_(2, 53)_ = 24.96, *p* < 0.001], but no significant group [*F*_(2, 53)_ = 1.07, *p* = 0.35], stimulus type [*F*_(1, 53)_ = 3.95, *p* = 0.052], spatial frequency by group [*F*_(4, 108)_ = 1.13, *p* = 0.35], stimulus type by group [*F*_(2, 53)_ =.65, *p* = 0.52], spatial frequency by stimulus type [*F*_(2, 53)_ = 0.09, *p* = 0.91], or stimulus type by spatial frequency by group effects [*F*_(4, 108)_ = 2.33, *p* = 0.06]. To follow up on the significant spatial frequency effect, we performed pairwise comparisons among spatial frequencies. Participants were significantly more accurate on LSF compared to NSF (mean difference = 1.0%, *p* < 0.001) or HSF (mean difference = 1.9%, *p* < 0.001) images, while they were significantly more accurate on NSF compared to HSF (mean difference = 0.9%, *p* = 0.007) images.

On the shapes stimuli, there were no significant stimulus type [*F*_(1, 54)_ < 1.27, *p* = 0.26], group [*F*_(2, 54)_ = 0.41, *p* = 0.66], or stimulus type by group [*F*_(2, 54)_ = 1.61, *p* = 0.21] effects on accuracy. See Table 2 for summary of behavioral results.

**Table 2:** **Accuracy and Reaction times for Face and House tasks, with control stimuli (shapes) as comparison**.

	**Anorexia Nervosa (AN)**	**Body Dysmorphic Disorder (BDD)**	**Healthy Controls (HC)**
Face Accuracy	95.7%	96.3%	96.8%
Face Reaction Time (ms)	855.2 ± 118.2	868.7 ± 106.8	800.3 ± 98.9
House Accuracy	95.9%	96.8%	96.8%
House Reaction Time (ms)	869.9 ± 164.0	837.7 ± 119.0	803.3 ± 119.3
Shape Accuracy	96.4%	95.5%	95.7%
Shape Reaction Time (ms)	769.9 ± 207.6	763.3 ± 72.9	746.0 ± 85.3

We also examined the effects of task behavioral performance on our ERP measures by testing correlations between reaction time and each measure (P100 amplitude, N170 amplitude, N170 latency). No correlations were significant at a corrected threshold of 0.05/3 = 0.016 (see Appendix, Table S1).

## ERP waveforms for both faces and houses are shown in Figure 2

### Electrophysiological results

#### P100 amplitude

We found a significant group effect [*F*_(2, 57)_ = 3.70, *p* = 0.031] and spatial frequency effect [*F*_(2, 56)_ = 8.70, *p* = 0.001]. (See Figure 3. Statistics for all main and interaction effects can be found in Table 3). To follow up the significant group effect, we performed group pairwise comparisons. The AN group had significantly lower amplitudes than BDD (mean difference = 1.48, *p* = 0.014) and controls (mean difference = 1.23, *p* = 0.04). The BDD group did not significantly differ from the controls (*p* = 0.66).

**Figure 2:**
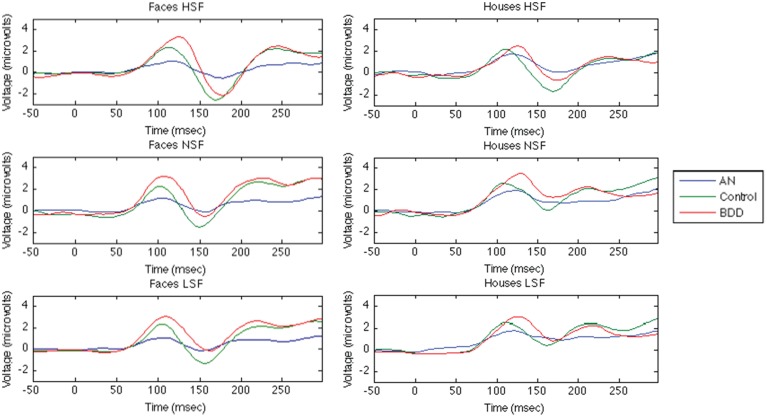
**Group· averaged ERP components for Face and House Tasks**. The first 50ms are the baseline period, stimulus presentation at time = 0.

**Figure 3:**
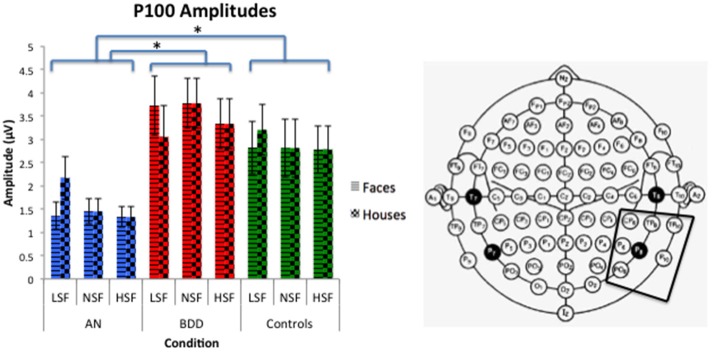
**P100 amplitudes across stimulus type (Faces/Houses), Group (AN, BDD, and Controls), and spatial frequency (LSF, Iow spatial frequency; NSF, normal spatial frequency; HSF, high spatial frequency)**. Asterisks denote significant group effects, *p* < 0.05, showing AN have smaller P100 amplitudes than BDD or controls for all image types.

**Table 3:** **Omnibus mixed measures ANOVA statistics for P100 Amplitude**.

**P100 Amplitude**	
**Effect**	**Statistics**
Main effect: group	*F*_(2, 57)_ = 3.70, *p* = 0.03
Main effect: stimulus type	*F*_(1, 57)_ = 0.09, *p* = 0.76
Main effect: spatial frequency	*F*_(2, 56)_ = 8.7, *p* = 0.001
Interaction effect: stimulus type × spatial frequency	*F*_(2, 56)_ = 2.81, *p* = 0.07
Interaction effect: stimulus type × group	*F*_(2, 57)_ = 1.71, *p* = 0.19
Interaction effect: group × spatial frequency	*F*_(4, 114)_ = 85, *p* = 0.50
Interaction effect: group × spatial frequency × stimulus type	*F*_(4, 114)_ = 21, *p* = 0.93

*Follow-up tests on the group effect found AN had significantly smaller amplitudes than controls (p = 0.014) and BDD (p = 0.04)*.

We investigated this further with a *post-hoc* analysis using time–frequency analysis, specifically event-related spectral perturbations (ERSP), to understand if differences in alpha or theta power or intertrial coherence could explain the AN amplitude difference. However, we did not find any significant differences between groups. Additionally, there were no differences in sleep or tiredness ratings. (See Appendix).

#### N170 amplitude

**Figure 4:**
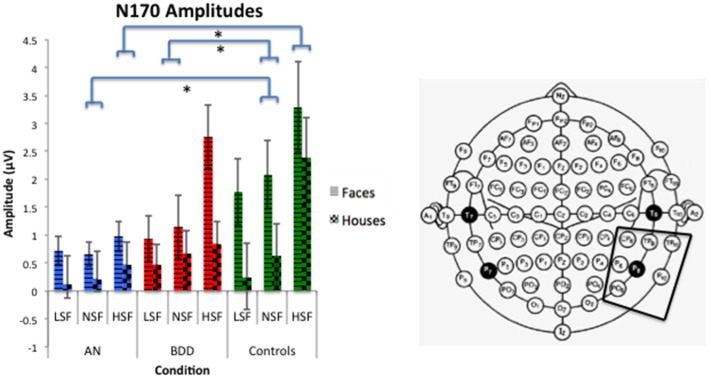
**N170 amplitudes across stimulus type (Faces/Houses), Group (AN, BDD, and Controls), and spatial frequency (LSF, Iow spatial frequency; NSF, normal spatial frequency; HSF, high spatial frequency)**. Asterisks denote significant group by spatial frequency effects, *p* < 0.05, showing AN have smaller N170 amplitudes than controls for HSF and NSF images, while BDD have smaller N170 amplitudes than controls for NSF images.

We found a significant group effect [*F*_(2, 57)_ = 3.74, *p* = 0.030], spatial frequency effect [*F*_(2, 56)_ = 34.24, *p* = 0.001], stimulus type effect [*F*_(1, 57)_ = 11.42, *p* = 0.001], and group by spatial frequency effect [*F*_(4, 114)_ = 3.54, *p* = 0.009]. (See Figure 4. Statistics for all main and interaction effects can be found in Table 4).

**Table 4:** **Omnibus mixed measures ANOVA statistics for N170 Amplitude**.

**N170 Amplitude**	
**Effect**	**Statistics**
Main effect: group	*F*_(2, 57)_ = 3.74, *p* = 0.03
Main effect: stimulus type	*F*_(1, 57)_ = 11.42, *p* = 0.001
Main effect: spatial frequency	*F*_(2, 56)_ = 34.24, *p* = 0.001
Interaction effect: stimulus type × spatial frequency	*F*_(2, 56)_ = 0.45, *p* = 0.64
Interaction effect: stimulus type × group	*F*_(2, 57)_ = 0.02, *p* = 0.56
Interaction effect: group × spatial frequency	*F*_(4, 114)_ = 3.54, *p* = 0.009
Interaction effect: group × spatial frequency × stimulus type	*F*_(4, 114)_ = 0.068, *p* = 0.41

*Follow-up tests on the group effect found AN had significantly smaller amplitudes than controls (p = 0.011) and BDD had a trend for smaller amplitudes than controls (p = 0.055). Follow-up tests on the group by spatial frequency effect found AN had significantly smaller amplitudes vs. controls for HSF (p = 0.001) and NSF (p = 0.034) images, while BDD had significantly smaller amplitudes vs. controls for NSF (p = 0.034) images*.

To follow up the significant group effect, we performed group pairwise comparisons. The AN group had significantly lower amplitudes than controls (mean difference = 1.32, *p* = 0.011), while the BDD group had a trend for lower amplitudes than controls (mean difference = 0.98, *p* = 0.055). The AN group did not significantly differ from the BDD group (*p* = 0.50).

To follow up on the significant group by spatial frequency effect, we performed group pairwise comparisons for each spatial frequency. The AN group had significantly lower N170 amplitudes for HSF and NSF images compared to controls (HSF: *p* = 0.001, NSF: *p* = 0.045), while the BDD group had significantly lower N170 amplitudes than controls for NSF images (*p* = 0.034).

#### N170 latency

**Figure 5:**
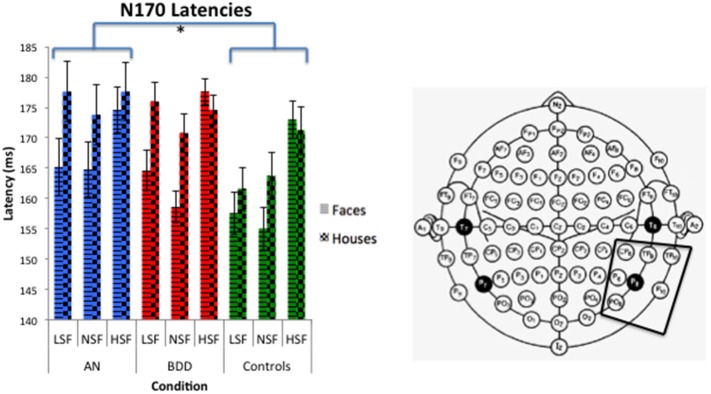
**N170 latencies across stimulus type (Faces/Houses), Group (AN, BDD, and Controls) and spatial frequency (LSF, Iow spatial frequency; NSF, normal spatial frequency; HSF, high spatial frequency)**. Asterisks denote significant group effects, *p* < 0.05, showing AN have significantly longer N170 latencies than controls for all image types.

We found a significant group effect [*F*_(2, 57)_ = 3.39, *p* = 0.041], stimulus effect [*F*_(1, 57)_ = 8.18, *p* = 0.006], spatial frequency effect [*F*_(2, 56)_ = 25.05, *p* = 0.001], and spatial frequency by stimulus type effect [*F*_(2, 56)_ = 8.013, *p* = 0.001]. (See Figure 5. Statistics for all main and interaction effects can be found in Table 5).

**Table 5:** **Omnibus mixed measures ANOVA statistics for N170 Latency**.

**N170 Latency**	
**Effect**	**Statistics**
Main effect: group	*F*_(2, 57)_ = 3.39, *p* = 0.041
Main effect: stimulus type	*F*_(1, 57)_ = 8.18, *p* = 0.006
Main effect: spatial frequency	*F*_(2, 56)_ = 25.05, *p* = 0.001
Interaction effect: stimulus type × spatial frequency	*F*_(2, 56)_ = 8.01, *p* = 0.001
Interaction effect: stimulus type × group	*F*_(2, 57)_ = 0.37, *p* = 0.69
Interaction effect: group × spatial frequency	*F*_(4, 114)_ = 1.44, *p* = 0.23
Interaction effect: group × spatial frequency × stimulus type	*F*_(4, 114)_ = 0.68, *p* = 0.61

*Follow-up tests on the group effect found that AN had significantly longer latencies than controls (p = 0.016) and BDD had a trend for longer latencies than controls (p = 0.059)*.

To follow up the significant group effect, we performed group pairwise comparisons. The AN group had significantly longer latencies than controls (mean difference = 8.57, *p* = 0.016), while the BDD group had a trend for longer latencies than controls (mean difference = 6.67, *p* = 0.059). The AN group did not significantly differ from the BDD (*p* = 0.585).

See Table 6 for all means and SEMs for P100 amplitudes, N170 amplitudes, and N170 latencies.

**Table 6:** **Means and SEMs for P100 amplitudes, N170 amplitudes, and N170 latencies**.

	**Spatial Frequency**	**Faces**			**Houses**		
		**AN**	**BDD**	**Controls**	**AN**	**BDD**	**Controls**
P100 mean amplitudes (μV)	LSF	1.35 ± 0.29	3.72 ± .63	2.82 ± 0.56	2.17 ± 0.46	3.05 ± 0.68	3.2 ± 0.54
	NSF	1.45 ± 0.27	3.77 ± .53	2.81 ± 0.63	1.45 ± 0.27	3.77 ± 0.53	2.81 ± 0.63
	HSF	1.33 ± 0.22	3.33 ± .53	2.78 ± 0.51	1.33 ± 0.22	3.33 ± 0.53	2.78 ± 0.51
N170 mean amplitudes (μV)	LSF	0.72 ± 0.25	0.93 ± 0.41	1.77 ± 0.59	0.12 ± 0.5	0.47 ± 0.37	0.25 ± 0.61
	NSF	0.66 ± 0.22	1.15 ± 0.57	2.08 ± 0.61	0.21 ± 0.51	0.67 ± 0.40	0.63 ± 0.58
	HSF	0.98 ± 0.27	2.76 ± 0.57	3.29 ± 0.81	0.47 ± 0.41	0.84 ± 0.41	2.39 ± 0.71
N170 mean latencies (ms)	LSF	165.2 ± 4.77	164.6 ± 3.30	157.6 ± 3.50	177.6 ± 4.94	176 ± 3.24	161.6 ± 3.56
	NSF	164.8 ± 4.55	158.6 ± 2.54	155 ± 3.53	173.8 ± 4.97	170.8 ± 3.24	163.8 ± 3.77
	HSF	174.6 ± 3.78	177.6 ± 2.16	173 ± 3.12	177.6 ± 4.74	174.6 ± 2.43	171.2 ± 3.94

### Correlations with clinical variables

**Figure 6:**
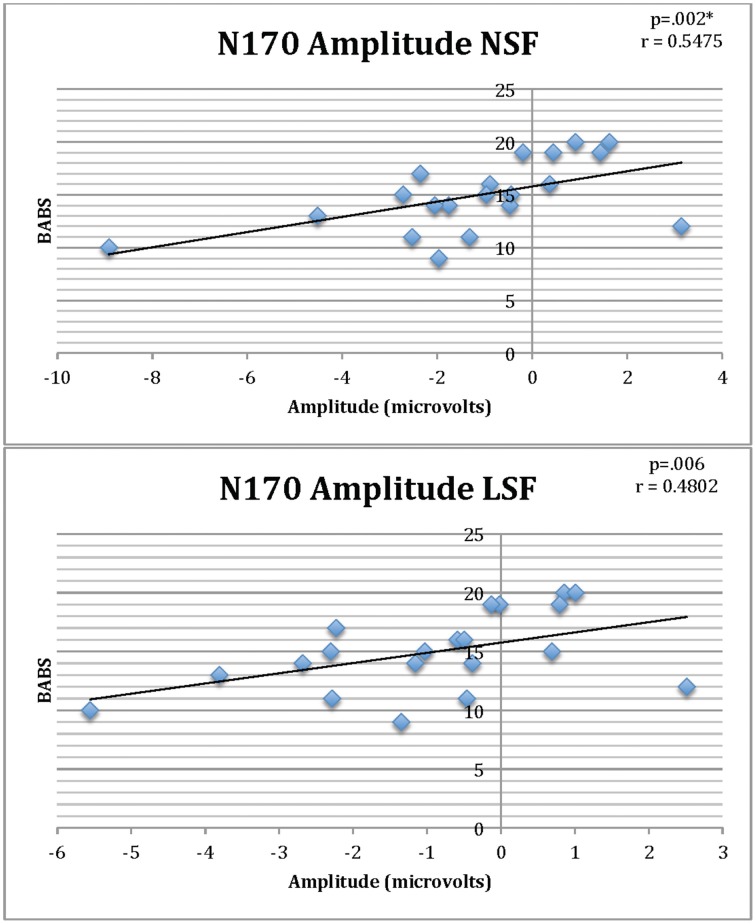
**Correlations of BABS scores and N170 amplitudes for NSF and LSF faces in individuals with BDD**. Lower (less negative) amplitude is associated with worse insight (higher BABS scores). ^*^Survives Bonferroni corrections for multiple comparisons.

There were significant positive correlations between BABS and N170 amplitudes for NSF (*r* = 0.54, *p* = 0.002) and LSF faces (*r* = 0.48, *p* = 0.006) in the BDD group, of which the former survived multiple comparisons (Figure 6) (There were no significant outliers as determined by leverage values).

## Discussion

This is the first EEG study in BDD, and the first study to investigate early visual processing components of P100 and N170 in either BDD or in AN. Results suggest that individuals with AN may have deficiencies in visual processing of configural information and enhanced detailed processing, as reflected in decreased P100 amplitudes and delayed N170 latencies, respectively. Because these abnormalities are evident irrespective of stimulus type or spatial frequency, they may be indications of general, early perceptual abnormalities. These effects are likely associated with low-level stimulus characteristics that are unrelated to appearance. Our results suggest that there may also be a similar deficiency in BDD, for which there was trend level significance for all three ERP measures, in the same direction as in AN. In the BDD group there is evidence for a relationship between diminished structural encoding of faces and greater perceptual distortions, as decreased N170 amplitudes on the faces task correlated with worse insight.

AN individuals showed significantly decreased P100 amplitudes compared to controls and BDD. They also showed significantly decreased N170 amplitudes compared to controls, specifically for high and normal spatial frequencies. In this time frame, this could represent an abnormality in very early configural processing, originating in dorsal extrastriate visual processing areas. This could explain the propensity of AN subjects to fixate on particular “fat” body parts at the exclusion of the whole, as well as the estimation of their size as larger than they actually are (Skrzypek et al., 2001). We found AN had a trend for greater event related desynchronization (ERD) compared to controls (See Appendix), which suggest AN could be compensating for their configural deficits through increased attention, effort, or arousal. AN individuals also had significantly delayed N170 latencies, which may be a reflection of enhanced detailed processing. Moreover, the fact that these abnormalities were evident across faces and houses suggests that this represents a general effect for all image types.

We also found that BDD, similar to AN, had significantly lower N170 amplitudes relative to controls for normal spatial frequency images. The BDD group additionally demonstrated significant positive correlations between N170 amplitude and BABS scores for NSF and LSF faces for BDD subjects, such that lower (less negative) amplitude is associated with worse insight (higher BABS scores). The BABS is a seven-item clinician administered interview that measures the amount of delusional thinking, belief, and insight in clinical populations. Thus, diminished N170 amplitudes, which could be a marker of abnormal structural encoding of faces, leading to an incomplete generation of a complete facial representation, which in turn contributes to perceptual distortions. Previous research found associations between higher BABS scores and low fractional anisotropy and high mean diffusivity of the inferior longitudinal fasciculus and forceps major (Feusner et al., 2014), as well as lower accuracy on the Navon task in global-local trials (Kerwin et al., 2014), suggesting a consistent association between neural and neuropsychological signatures and poor insight across several studies in BDD.

Previous neuropsychological and neuroimaging studies on visual/visuospatial processing in AN and BDD suggest imbalances in detail and configural processing. Superior attention to detail and poor central coherence compared with controls was observed in both active and recovered AN participants (Tenconi et al., 2010; Roberts et al., 2013). A pattern in the current study of delayed N170 latencies for AN support previous findings of weak central coherence in AN (Smeets et al., 1999; Lopez et al., 2008, 2009; Kim et al., 2011). Our results provide a better estimation of when these abnormalities may occur, as we see differences as early as 100 ms after stimulus presentation.

Previous ERP studies in AN using visual stimuli focused on later components associated with emotional responses (Dodin and Nandrino, 2003; Pollatos et al., 2008). One study found that individuals with AN had larger amplitude and longer latency P300s in response to body images, interpreted as hyperarousal in information processing (Dodin and Nandrino, 2003), whereas another that focused on N200 and P300 signals in response to emotional faces found abnormalities in emotional processing (Pollatos et al., 2008).

If these findings of abnormal ERP patterns are replicated in future studies, they have the potential to provide useful biomarkers that could be translated to clinical use. If this is the case, they may be more practical than biomarkers from neural patterns generated from fMRI experiments, due to lower cost, higher temporal resolution, and efficiency of EEG. These biomarkers of early visual components would be advantageous because they are less likely to be affected by emotional, subjective, or motivational factors, relative to psychometric measurements, and potentially provide a dimensional “bio-signature” of an important phenotype shared by AN and BDD. Findings from this study would therefore have relevance for informing the development of treatments to address perceptual distortions such as perceptual retraining; these would require different strategies depending on the pathophysiological mechanism driving the symptoms. In addition, these markers can be monitored over time to assess treatment efficacy in common practices to treat these disorders such as cognitive behavioral therapy. In previous studies, EEG and ERPs have been used as biomarkers for Alzheimer's and Mild Cognitive impairment (MCI) (Jackson and Snyder, 2008) as well as for detecting early visual processing deficits in schizophrenia (Knebel et al., 2011). Thus, abnormalities, or brain-behavior relationships, in P100 or N170 components in AN or BDD could serve as trait markers underlying their visual processing deficits; this could lead to more accurate prediction of AN or BDD risk and diagnosis. In addition, since persistent perceptual disturbance has been found to be a strong predictor of relapse in AN and bulimia nervosa (Keel et al., 2005), it could potentially also be used prognostically.

This study has several limitations. The recordings are observed at the scalp level, so we cannot specify exactly which cortical regions are dysfunctional in these subjects. We also investigated weight-restored AN participants, so results may not be able to be generalized to individuals in the underweight state. Moreover, we cannot determine if effects in the current study are the result of their previous starvation state. Because it is difficult for weight-restored individuals with AN to estimate the previous duration of time they were in the underweight state, we did not have this data available for regression analyses. (Although we examined correlations with lowest BMI attained, we found no significant associations.) In the future, a longitudinal study could track this with better precision in order to explore relationships with duration of starvation state.

Future studies can use source analyses in conjunction with ERPs to further localize, with more precision, abnormalities in the brain. In addition, since images of faces and houses could still elicit higher level processing from emotion or memory areas, using stimuli such as Gabor patches could be more fruitful to investigate lower-level visual processing. Furthermore, joint analyses integrating different neuroimaging modalities such as fMRI or sMRI can enable inferences to be made on both hemodynamic and electrical sources of neural activity.

In the interest of classifying psychopathology across multiple domains of analysis, our results suggest an electrophysiological underpinning behind the symptoms of distorted body image in AN and BDD. These can form the basis of additional dimensions by which we can understand these various disorders that can be used in conjunction with current psychometric and behavioral methods. As a result, new treatments may be developed based on the mechanisms underlying the symptoms; for example, perceptual retraining with visual stimuli may be more effective for individuals with more severe visual distortions. Thus, this data can be used both as biomarkers of abnormal visual processing and to provide a deeper understanding of abnormal brain activation patterns in these disorders involving body image.

### Conflict of interest statement

This study was funded by grant NIH MH093535-02S1 (Feusner) and a grant from the International OCD Foundation (Li). All authors report no biomedical financial interests or potential conflicts of interest. The authors declare that the research was conducted in the absence of any commercial or financial relationships that could be construed as a potential conflict of interest.

## Appendix

### ERSP: event related synchronization and desynchronization

To complement our event related potential (ERP) analyses, we also performed time-frequency analyses using event related spectral perturbances (ERSP) to characterize ongoing EEG rhythmic activity and their frequency power spectrum over time. ERSPs provide information about modulations of oscillatory activity not phase-locked to the stimulus, as are ERPs (Pfurtscheller and Lopes da Silva, 1999). We chose to examine event-related synchronization (ERS) and desynchronization (ERD) in the alpha (8–12 Hz) and theta (4–7 Hz) ranges during the first 200 ms post-stimulus; ERD and ERS are considered to be due to decreases or increases in synchrony of the underlying neuronal populations, respectively (Pfurtscheller and Lopes da Silva, 1999). During visual processing, alpha and theta normally respond in opposite ways; theta synchronizes while alpha desynchronizes, relative to baseline (Pfurtscheller et al., 1996). The alpha ERD (and corresponding theta ERS) is thought to be a reflection of activation of cortical areas related to sensory processing, and could be the result of increased attention, effort, or arousal (Pfurtscheller and Lopes da Silva, 1999). We used these frequency measures to better understand the effects of these cognitive and attentional variables on the early visual processing occurring in AN and BDD.

We also used intertrial coherence (ITC) to measure the consistency of phase across trials at each frequency and time point (Makeig et al., 2002). In this way, we are able to investigate any abnormalities in phase synchronization or phase consistency across trials in AN and BDD.

### Methods

Single trials (from 0.5 s before stimulus to 2 s after) were convolved time locked to a complex morlet wavelet, setting the number of cycles to be 3 in 1 s. This resulted in 3 Hz as the lowest frequency analyzed. Spectral power modulations were measured relative to a baseline of 200 ms pre-stimulus. We collapsed all three spatial frequencies for these analyses, as we did not have any a priori hypotheses about spatial frequency, and to increase number of trials for the analysis. Comparisons were performed using averaged power or ITC values from time-frequency windows chosen around the frequency and time ranges of interest (Alpha: 8–12 Hz, Theta: 4–7 Hz, P100: 72–128 ms, N170: 140–196 ms). We performed a Four-Way ANOVA, with Group (AN, BDD, controls), Electrode (Oz, Pz), Frequency (Alpha 8–12 Hz, Theta 4–7 Hz), and Component (P1, N170) as factors. Significant ANOVAs were submitted to *post-hoc* One-Way ANOVAs and Tukey tests to compare pairwise effects of interest.

### Results

We found a trend for a group by frequency interaction effect [*F*_(2, 471)_ = 2.745, *p* = 0.065] for the faces task. *Post-hoc* univariate ANOVAs performed on the individual frequencies showed a trend for a significant difference in the alpha range [*F*_(2, 235_ = 3.017, *p* = 0.051]. Pairwise comparisons found that this was driven by AN having greater alpha desynchronization compared with controls (*p* = 0.041).

We did not find any significant differences in ITC between groups.

### Correlations with face ratings

Based on the group results findings, we performed *post-hoc* Pearson correlation analyses within the AN group between alpha power and participants' subjective ratings of the attractiveness, aversiveness, and degree to which thoughts of self were triggered when viewing the face stimuli (collected after the experiment). There were no significant outliers as determined by leverage values. We found a significant positive correlation between alpha power and aversiveness ratings (*r* = 0.67, *p* = 0.017). Thus, lower alpha power (which occurs with desynchronization and may reflect increased attention, effort, or arousal) is associated with lower subjective aversiveness of the face.

### Correlations with sleep and tiredness ratings

We also performed post-hoc Pearson correlation analyses on the significant measures from the ERP analyses (N170 Amplitude Faces HSF, N170 Latency Houses LSF, P100 Amplitude Faces LSF, P100 Amplitude Faces NSF, and P100 Amplitude Faces HSF) with sleep and tiredness ratings for AN and BDD groups. However, we found no significant associations.

### Correlations with reaction time

To investigate the effects of variations of reaction time on ERP measures, we performed correlations with each EEG measure (P100 amplitude, N170 amplitude, N170 latency) and the corresponding reaction times. We found no significant correlations at corrected threshold of .05/3 = 0.016.

**Table S1:**

		**RT**
**P100_AMP**	Pearson Correlation	−0.061
	Sig. (2-tailed)	0.250
	N	356
**N170_AMP**	Pearson Correlation	−0.101
	Sig. (2-tailed)	0.057
	N	356
**N170_LAT**	Pearson Correlation	−0.014
	Sig. (2-tailed)	0.796
	N	356
